# Impact of a lifestyle education program on family medicine residents’ knowledge and attitudes: A pre- and post-intervention study

**DOI:** 10.3389/fpubh.2026.1817798

**Published:** 2026-06-19

**Authors:** Amal J. Alfaifi, Mohammad Altherwi, Musa M. Altherwi, Hamoud Elneami, Abdulrahman Y. Sumayli, Sulaiman Hamdi, Ali Ibrahim Ali, Ibrahim M. Gosadi, Majed A. Ryani, Ayman Afify Konswa

**Affiliations:** 1 Family Medicine Department, Jazan Health Cluster, Jazan, Saudi Arabia; 2 Department of Family and Community Medicine, College of Medicine, Jazan University, Jazan, Saudi Arabia; 3 Prince Sultan Military Hospital, Riyadh, Saudi Arabia

**Keywords:** family medicine, intervention, Jazan, lifestyle medicine, medical education, Saudi Arabia

## Abstract

**Background and objectives:**

Non-communicable diseases are leading causes of morbidity and mortality worldwide. Lifestyle behaviors, such as poor diet, physical inactivity, and tobacco use, contribute substantially to this burden. Lifestyle medicine (LM) emphasizes evidence-based behavioral interventions to prevent and manage chronic diseases. However, little is known about LM education among family medicine residents in Saudi Arabia, particularly in the Jazan region. This study aimed to assess the effects of a structured LM educational intervention on the knowledge and attitudes of family medicine residents in the Jazan region.

**Methods:**

A pilot pre- and post-intervention design was implemented among 67 residents enrolled in a post-graduate family medicine program in the Jazan region during May 2025. A validated questionnaire covering eight knowledge domains and five attitude dimensions related to LM was administered before and after training. The intervention was performed in a single day. An 8-h intensive program provided a comprehensive introduction to LM and health coaching through a blended learning approach incorporating lectures, workshops, and case-based scenarios. Facilitated by International Board of Lifestyle Medicine-certified consultant family physicians, the curriculum leveraged expert instruction and multimedia tools to deliver evidence-based training on the Six Pillars of Lifestyle Medicine. Data were analyzed using descriptive and comparative statistics to evaluate the changes between pre- and post-intervention responses.

**Results:**

The median total knowledge score increased from 23.0 (IQR 19.0–29.0) at baseline to 29.0 (IQR 27.0–31.0) following the intervention. Knowledge improved across the majority of domains, notably nutrition, physical activity, and health coaching. Knowledge improved among 82.1% of participants. A total of 56.7% demonstrated a positive shift in attitudes toward LM principles, which may translate into greater willingness to counsel patients on lifestyle modification and prioritize preventive care during routine clinical encounters.

**Conclusion:**

The educational intervention significantly improved residents’ knowledge and moderately enhanced their attitudes toward LM. The findings of this pilot study underscore the importance of incorporating structured LM training into family medicine residency programs nationwide to strengthen preventive care competencies and support the goals of Saudi Vision 2030.

## 1 Introduction

Non-communicable diseases (NCDs) are long-term health conditions caused by a combination of genetic, physiological, environmental, and behavioral factors, including illnesses such as heart attacks, strokes, cancers, and chronic respiratory disorders such as asthma, chronic obstructive pulmonary disease, and diabetes. NCDs can affect individuals of all ages and exist in every country around the world. Although these illnesses are linked to older adults, 18 million NCD-related deaths occur before the age of 70. NCDs are the leading cause of death in this age group than any other causes. Approximately 82% of premature deaths occur in low- and middle-income countries. Several behavioral factors increase the risk of NCDs, including tobacco use, unhealthy diets, excessive alcohol consumption, and physical inactivity. These behaviors can cause metabolic changes, such as high blood pressure, obesity, diabetes, and high cholesterol levels. Elevated blood pressure levels account for 25% of global NCD fatalities, followed by high blood glucose levels, overweight, and obesity.

In primary care, high-impact NCD interventions can improve early detection and management (1). Saudi Arabia mirrors this global trend: NCDs account for approximately 73% of all deaths, with a probability of premature mortality of approximately 18.6%. Modifiable behaviors, including tobacco use, unhealthy diets, physical inactivity, and obesity, have contributed to NCD-related deaths in Saudi Arabia, rising from 65% of the total mortality rate in 2000 to 73% in 2016 (2). National surveillance data show that risk factors for NCD are common in Saudi Arabia. The World Health Organization’s NCD country profile reported that 52% of Saudi adults were insufficiently active, with a higher proportion of inactivity among women (64%) compared to men (44%); obesity affects 35% of adults, with a greater prevalence in women (41%) than in men (31%) (3). The 2019 World Health Survey further supported these data, revealing that 80% of respondents were not active enough (4). Specifically, in the Jazan region, the prevalence of diabetes mellitus was 12.3% (5). Furthermore, primary health care centers in the region have reported that NCDs account for approximately 78% of mortality rates, with physical inactivity and unhealthy lifestyles identified as important risk factors (6). These findings emphasize the importance of addressing lifestyle factors in primary health care (PHC). Additionally, official consensus statements from the American College of Lifestyle Medicine Experts emphasize the importance of integrating lifestyle medicine (LM) practice into primary care to ensure the quality of care delivered, inform policy decisions, and suggest areas for future research (7).

The American Board of Lifestyle Medicine defines LM as a branch of medicine that uses evidence-based approaches to reverse and prevent lifestyle-related chronic diseases, such as type 2 diabetes, obesity, and cardiovascular disease. It focuses on six interrelated lifestyle areas: nutrition, physical activity, sleep, stress management, social support, and avoiding risky substances. Family medicine is an ideal setting for LM because it allows for continuous care, long-term patient relationships, and regular visits (8). A recent study of 14 LM training programs for health professionals found major improvements in three areas: knowledge (SMD = 0.71; 95% CI = 0.25–1.18), self-confidence in counseling (SMD = 1.34; 95% CI = 0.61–2.07), and the adoption of healthy habits or changes in practices (SMD = 0.78; 95% CI = 0.29–1.26) (8). These results indicate that LM training not only transfers knowledge but also enhances physicians’ personal health behaviors and equips them with valuable clinical counseling skills.

The meta-analysis revealed that the majority of studies did not measure attitudes in a way that could be combined, indicating limited data on how LM education affects attitudes toward preventive care (9). This evidence suggests that while LM training can improve health professionals’ counseling, further research is needed to study how it changes their attitudes and long-term habits. Saudi Arabia’s Health 2030 health sector transformation program prioritizes LM. Strengthening primary healthcare and preventing NCDs are key objectives for improving public health and managing chronic diseases (10). The Saudi Commission for Health Specialties (SCFHS) launched a 2022 training fellowship in LM. It emphasizes the resolution of prevailing clinical and community health problems and identifies prevention, patient engagement, and behavioral counseling as core components of the family medicine training program (10, 11). National programs addressing nutrition, physical activity, and tobacco control have been launched. The ongoing high mortality rates and prevalence of risk factors associated with NCDs indicate that the current implementation of lifestyle interventions is incomplete, and primary care providers require training to effectively deliver these interventions.

Although national policies support preventive health and LM skills, there is very little published research on LM training in Saudi family medicine residencies. A recent nationwide study of medical schools revealed that no research has examined instructional methods for all components of the LM curriculum in Saudi Arabia (12). There are no published before-and-after studies on LM interventions among family medicine residents in the Jazan region. Because NCDs are a major problem in the region, and PHCs can help with prevention, this lack of training is a serious problem. Therefore, this study used a pilot pre- and post-intervention to determine how a specific LM training module affected family medicine residents in Jazan. The primary outcome will be changes in residents’ knowledge of and attitudes toward lifestyle medicine. The intervention aimed to promote the preventive goals of Saudi Vision 2030 and meet the rising expectations of SCFHS for family medicine graduates. We will achieve this by enhancing residents’ skills in evidence-based LM through training residency programs at a wider national level.

## 2 Materials and methods

### 2.1 Study design and settings

This pilot pre- and post-intervention study targeted a postgraduate family medicine residency program in Jazan city. The intervention was performed in May 2025. Family medicine residents in the postgraduate family medicine program in Jazan city, including all residents at levels R1, R2, and R3, participated in the study.

### 2.2 Data collection tool

The questionnaire used in the current investigation was adapted from a previous study by Alzaben et al. (13), who developed a questionnaire to evaluate knowledge, attitudes, and practices in LM domains. The questionnaire was designed to measure the incorporation of LM into routine clinical work and medical education programs among medical students and medical staff. Alzaben et al. conducted a validity assessment using face and content validity and assessed internal consistency using Cronbach’s alpha test (13).

To meet the goals of the current investigation, the questionnaire comprised three main sections: demographic, LM knowledge, and attitudes toward LM practice in family medicine clinics. The first was modified to meet the training background of the targeted family medicine residents and to assess their sociodemographic and clinical characteristics. The second part, related to knowledge, was divided into eight domains: the first domain, related to nutrition, included five questions, and the second domain, related to physical activity, included five questions. The third domain, related to sleep health, included five questions, and the fourth domain, related to smoking, included five questions. The fifth domain, related to alcohol control, included five questions. The sixth domain of health and wellness coaching includes five questions, while the seventh domain of weight management includes two. The second part of the questionnaire on attitudes included five questions using a response scale from strongly disagree to strongly agree. Internal consistency of the utilized tool was tested via Cronbach’s alpha test, revealing a value of 0.816, indicating good reliability among the current sample.

### 2.3 Data collection process

A team of trained research assistants conducted interviews. Before data collection, all research assistants participated in a standardized training session. This training included a detailed review of the questionnaire, a discussion of ethical conduct, and a demonstration of how to approach participants respectfully and obtain informed consent. Standardized scripts and guidelines were used to ensure a consistent approach across all interviews, thereby minimizing interviewer-related bias. To ensure data integrity and prevent duplicate or overlapping responses, several measures were implemented. A recruitment log with the participants’ email addresses was maintained to prevent duplicate enrollment at all residency levels and to ensure the same participant received the same response before and after the intervention. Research assistants verified at the time of initial contact that participants had not previously completed the survey.

A total of 67 residents were included in the current analysis. This constituted the total number of trainees enrolled in the targeted family medicine residency program. Given the pilot nature of the current investigation, a convenience sampling approach was utilized, and all trainees were approached without a formal priori power calculation. This was necessary to assess intervention feasibility, curriculum appropriateness and comprehensiveness, and to generate effect size estimates. Therefore, the findings of the current analysis are treated as an educational efficacy rather than definitive hypothesis testing.

### 2.4 The intervention

The intervention consisted of a single-day, 8-h (8:00 a.m.–4:00 p.m.) intensive training program based on the Lifestyle Medicine Handbook (4th Edition) from the American College of Lifestyle Medicine (ACLM). Conducted on-site in a dedicated training lecture hall, the curriculum utilized a blended learning format structured into five lectures: an introduction to LM, a session on health coaching and behavior change (incorporating two interactive workshops), and three lectures covering the Six Pillars of LM in depth. Delivery methods combined didactic lectures for core theory, case-based learning involving clinical scenarios from family medicine, and multimedia instructional videos focusing on coaching techniques. The program was facilitated by two Consultant Family Physicians holding certificates from the International Board of Lifestyle Medicine and formal Training of Trainers accreditation. These facilitators are active members of the ACLM and accredited residency trainers, ensuring the intervention was delivered by experts specifically qualified in both the subject matter and postgraduate medical education.

### 2.5 Data analysis

Data editing was performed after exporting the responses to a Microsoft Excel sheet, including coding of open-ended responses. Incomplete questionnaires were excluded from the final analysis. Furthermore, during the data-cleaning process, we found a minimal number of missing values for specific questions. These missing values were not imputed and were handled by listwise deletion during the statistical analysis. This was applied to two measured demographic variables among two participants only.

Statistical analysis was performed using SPSS. The data analysis used both descriptive and difference between pre- and post-intervention, categorical data use frequency and percentage, each category in the questionnaire calculated the total for pre-and post-intervention, and measured the difference between pre-and post-intervention.

Following the descriptive analysis, inferential analysis was performed to assess the presence of a statistically significant difference in levels of knowledge and attitude before and after the implementation of the educational intervention. After the assessment of data distribution and given the presence of skewed findings, non-parametric tests were selected. For the current analysis, the Wilcoxon signed-rank test was used to compare the level of knowledge and attitude before and after the implementations within the measured domains. A *p*-value of 0.05 or less was considered statistically significant for the applied statistical tests.

**Figure 1:**
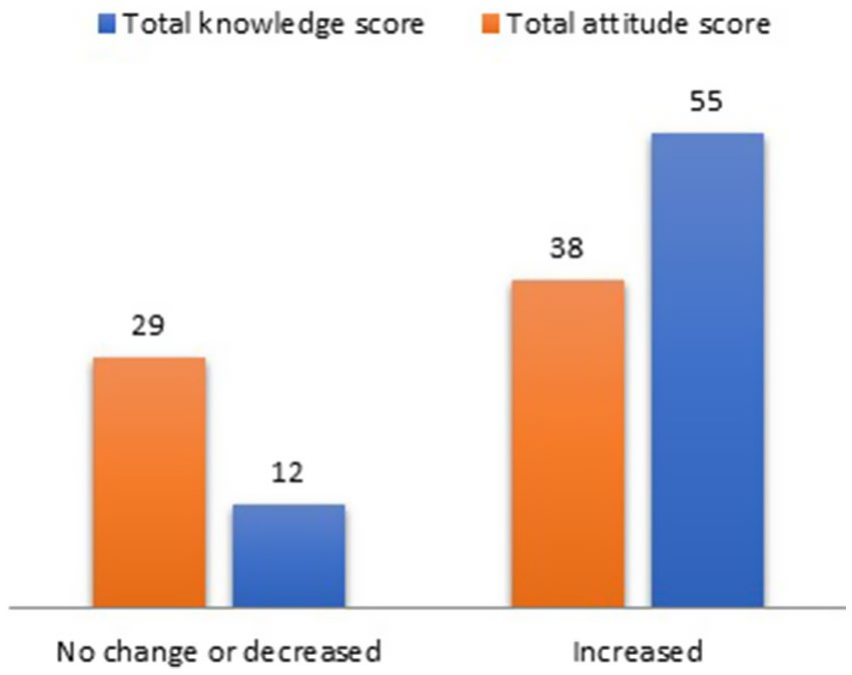
The study evaluated a total cohort of 67 participants to assess the impact of the educational intervention on domain-specific knowledge and attitudes. Post-intervention outcomes revealed a substantial improvement in cognitive metrics, yields a knowledge acquisition success rate of 82.1% (n = 55), whereas only 17.9% (n = 12) of the sample exhibited static or declined knowledge scores. Conversely, affective metrics demonstrated a more modest shift; the attitude improvement rate reached 56.7% (n = 38), leaving a considerable 43.3% (n = 29) classified as non-responders who demonstrated no change or a decrease in their total attitude scores. Collectively, these findings suggest that while the instructional design was highly effective for factual knowledge transfer, it proved noticeably less potent in modifying participants’ underlying perspectives or professional mindsets.

### 2.6 Ethical considerations

The study protocol was approved by the Ethics Committee of Jazan Health Cluster (approval number: 24116). Digital informed consent was obtained from all participants.

The informed consent form was provided in English, and contained details of the study’s purpose, procedures, potential risks and benefits, and the voluntary nature of participation. Each participant was a family medicine resident who understood the English language. Digital consent was obtained by the research assistants who orally explained all aspects of the study to the participants before they provided their consent.

All collected data were anonymized by assigning a unique identification number to each participant to ensure that their personal information was not linked to their responses. Digital data were stored in a password-protected file on a secure computer, accessible only by the research team. Not all identifying information was collected from participants except registered email to link pre- and post-responses for the same participant.

## 3 Results

Table 1 represents the demographic distribution of the participants. A total of 67 participants were included in the study. The majority were males (59.7%), while females accounted for 40.3%. Regarding age, 50.7% were 29 years or older, and 49.3% were younger than 29; based on body mass index (BMI), 58.2% were of normal weight, 34.3% were overweight or obese, and 7.5% were missing BMI data. According to residency level, 37.3% of participants were R1, 34.3% were R2, and 28.4% were R3. The majority (85.1%) had never smoked, and only 4.5% recorded that they currently smoked. In regard to exercise, 41.8% were not exercising while 58.2% exercised regularly 1 to 5 days a week; of those who exercised, session duration was less than 30 min for 20.9%, 30 min to 1 h for 25.4% and more than 1 h for 19.4%; for activity types, *sometimes* performing aerobic exercise was most common with 49.3%, while only 14.9% reporting *always*. Muscle strengthening exercise was *sometimes* 28.4% and *always* 14.9%. Dietary habits showed consumption *sometimes* predominated whole grain 68.7%, fruit and vegetables 59.7%, low-fat meat 52.2%, low-fat products 50.7%, and avoiding sugary foods 43.3%. Most participants (58.2%) slept 5–7 h per day, 31.3% slept more than 8 h, and only 10.4% slept less than 5 h.

**Table 1:** Sociodemographic and lifestyle characteristics of family medicine residents (*n* = 67).

Variable	*N* (%)
Age, years	
<29	33 (49.3)
≥29	34 (50.7)
Body mass index category	
Normal weight	39 (58.2)
Overweight or obese	23 (34.3)
Missing	5 (7.5)
Gender	
Male	40 (59.7)
Female	27 (40.3)
Year of residency training	
R1	25 (37.3)
R2	23 (34.3)
R3	19 (28.4)
Smoking status	
Current smoker	3 (4.5)
Former smoker	6 (9.0)
Passive smoker	1 (1.5)
Never smoked	57 (85.1)
Exercise frequency, days per week	
0 (no exercise)	28 (41.8)
1 day	6 (9.0)
2 days	10 (14.9)
3 days	12 (17.9)
4 days	4 (6.0)
5 days	7 (10.4)
Exercise duration per session, minutes	
No exercise	23 (34.3)
< 30 min	14 (20.9)
30–59 min	17 (25.4)
≥ 60 min	13 (19.4)
Aerobic exercise participation	
Always	10 (14.9)
Often	10 (14.9)
Sometimes	33 (49.3)
Rarely/almost never	3 (4.5)
Never	11 (16.4)
Muscle-strengthening exercise participation	
Always	10 (14.9)
Often	9 (13.4)
Sometimes	19 (28.4)
Rarely/almost never	9 (13.4)
Never	20 (29.9)
Whole-grain consumption	
Always	4 (6.0)
Often	12 (17.9)
Sometimes	46 (68.7)
Rarely/almost never	3 (4.5)
Never	2 (3.0)
Fruit and vegetable intake	
Always	5 (7.5)
Often	10 (14.9)
Sometimes	40 (59.7)
Rarely/almost never	7 (10.4)
Never	5 (7.5)
Low-fat meat choices	
Always	8 (11.9)
Often	12 (17.9)
Sometimes	35 (52.2)
Rarely/almost never	7 (10.4)
Never	5 (7.5)
Low-fat product choices	
Always	5 (7.5)
Often	14 (20.9)
Sometimes	34 (50.7)
Rarely/almost never	8 (11.9)
Never	6 (9.0)
Avoiding sugary foods	
Always	9 (13.4)
Often	20 (29.9)
Sometimes	29 (43.3)
Rarely/almost never	7 (10.4)
Never	2 (3.0)
Sleep duration, hours per night	
<5 h	7 (10.4)
5–7 h	39 (58.2)
≥8 h	21 (31.3)

Table 2 compares participants’ health knowledge median scores on multiple domains before and after the intervention. Improvement was observed in nutrition from 3.00 [2.00–4.00] pre-intervention to 4.00 [3.00–5.00] post-intervention, physical activity from 4.00 [3.00–5.00] to 5.00 [4.00–5.00], and general health and wellness coaching knowledge from 1.00 [1.00–2.00] to 3.00 [2.00–4.00]. Sleep, smoking, emotion and mind, and weight management remained unchanged: sleep from 4.00 [2.00–5.00] to 4.00 [4.00–5.00], smoking from 4.00 [3.00–5.00] to 4.00 [4.00–5.00], emotion and mind from 4.00 [2.00–4.00] to 4.00 [4.00–5.00], weight management from 2.00 [1.00–2.00] to 2.00 [2.00–2.00]. Alcohol slightly decreased from 3.00 [2.00–3.00] to 2.00 [2.00–3.00]; however, the total health knowledge median score increased from 23.00 [19.00–29.00] pre-intervention to 29.00 [27.00–31.00] post-intervention. All of the changes were statistically significant (*p*-values less than 0.05) except for the change in smoking cessation knowledge (p-value: 0.09) and alcohol control knowledge (0.369).

**Table 2:** Median lifestyle medicine knowledge scores before and after the educational intervention (*n* = 67).

Domain	Pre-intervention median (IQR)	Post-intervention median (IQR)	*p* value
Nutrition knowledge	3.00 (2.00–4.00)	4.00 (3.00–5.00)	<0.001
Physical activity knowledge	4.00 (3.00–5.00)	5.00 (4.00–5.00)	<0.001
Sleep health knowledge	4.00 (2.00–5.00)	4.00 (4.00–5.00)	<0.001
Smoking cessation knowledge	4.00 (3.00–5.00)	4.00 (4.00–5.00)	0.09
Alcohol control knowledge	3.00 (2.00–3.00)	2.00 (2.00–3.00)	0.396
Emotional and mental health knowledge	4.00 (2.00–4.00)	4.00 (4.00–5.00)	0.002
General health and wellness coaching knowledge	1.00 (1.00–2.00)	3.00 (2.00–4.00)	<0.001
Weight management knowledge	2.00 (1.00–2.00)	2.00 (2.00–2.00)	0.009
Total knowledge score	23.00 (19.00–29.00)	29.00 (27.00–31.00)	<0.001

In general, median scores increased across several lifestyle health knowledge domains, indicating improved participants’ health knowledge after the intervention. Nonetheless, the reduction of knowledge pertaining to the alcohol domain can be partially explained by the limited practical implications of alcohol control in family medicine clinics in a Saudi Arabian context, as alcohol consumption is not allowed in the country. This is likely to have impacted the ability of the trainers to deliver practical real-life case studies within the training course to the attendees and may have reduced the effectiveness of raising knowledge within the alcohol control domain.

Table 3 compares participants’ median health attitude scores across multiple domains before and after the intervention. There was an increase in median scores in nutrition from 19.00 [16.00–21.00] to 21.00 [16.00–23.00] post intervention, physical activity from 19.00 [16.00–21.00] to 21.00 [17.00–23.00], sleep from 19.00 [16.00–21.00] to 21.00 [16.00–23.00], smoking from 19.00 [16.00–22.00] to 21.00 [17.00–23.00], alcohol from 19.00 [16.00–21.00] to 21.00 [16.00–23.00], emotion and mind from 18.00 [16.00–21.00] to 19.00 [17.00–22.00], and weight management from 20.00 [16.00–21.00] to 21.00 [16.00–23.00]. The scores for general health and wellness coaching showed no notable change at 18.00 [16.00–21.00] before and 18.00 [16.00–22.00] after the intervention. However, the total health attitudes median score increased from 171.00 [144.00–189.00] pre-intervention to 180.00 [150.00–199.00] post-intervention. All the changes are statistically significant across all attitude domains (*p*-values < 0.05). These findings suggest a significant positive impact on participants’ health attitudes across most lifestyle domains following the intervention.

**Table 3:** Median lifestyle medicine attitude scores before and after the educational intervention (*n* = 67).

Domain	Pre-intervention median (IQR)	Post-intervention median (IQR)	*p*-value
Nutrition	19.00 (16.00–21.00)	21.00 (16.00–23.00)	0.006
Physical activity	19.00 (16.00–21.00)	21.00 (17.00–23.00)	0.031
Sleep health	19.00 (16.00–21.00)	21.00 (16.00–23.00)	0.025
Smoking cessation	19.00 (16.00–22.00)	21.00 (17.00–23.00)	0.007
Alcohol use	19.00 (16.00–21.00)	21.00 (16.00–23.00)	0.036
Emotional and mental health	18.00 (16.00–21.00)	19.00 (17.00–22.00)	0.024
Mindfulness	18 (16.00–20.00)	18 (16.00–22.00)	0.011
Health promotion	18.00 (16.00–21.00)	18.00 (16.00–22.00)	0.012
Weight management	20.00 (16.00–21.00)	21.00 (16.00–23.00)	0.044
Total attitude score	171.00 (144.00–189.00)	180.00 (150.00–199.00)	0.010

Table 4 presents the overall change in participants’ knowledge and attitudes after the intervention 55 (82.1%) participants showed an increase in their total knowledge score, while only 12 (17.9%) showed a reduction or no change; regarding attitudes, 38 (56.7%) participants showed improvement in their total attitudes score, and 29 (43.3%) showed a reduction or no change. These findings show that the intervention generally had a positive impact on participants’ knowledge and attitudes (see Figure 1).

**Table 4:** Changes in total knowledge and attitude scores following the intervention.

Items	Increased, *n* (%)	No change or decrease, *n* (%)
Total knowledge score	55 (82.1)	12 (17.9)
Total attitude score	38 (56.7)	29 (43.3)

## 4 Discussion

This pilot pre- and post-investigation study targeted family medicine residents in the postgraduate family medicine program in the Jazan region of Saudi Arabia to assess the effects of a structured LM educational intervention on the knowledge and attitudes of family medicine residents in the Jazan region. The findings indicated an overall improvement in knowledge and attitude of the participants among most measured domains. However, the improvement was more noticeable among the knowledge domains in comparison to the attitude domains. This indicates that despite the positive change in the knowledge of the residents concerning LM medicine components, the limited improvement in their attitude might indicate the presence of resistance to change, suggesting the need for further assessment of factors associated with it. Furthermore, this might be an area for improvement in the designed intervention: focus on techniques and strategies to initiate change in attitude of the physicians and, in turn, their subsequent adoption of LM practices in their clinical settings, rather than mainly on enhancing knowledge domains.

The findings of the current study can be compared to similar national or international investigations. A systematic review and meta-analysis conducted by Ibrahim et al (9). to assess the effectiveness of LM educational interventions on knowledge, competence, confidence, and skills among health professionals who belong to different specialties, such as nursing, physician assistants, and students (8). The systematic review by Ibrahim et al. included 14 interventional studies, most of which were from American or European contexts, with two from Hong Kong and one from Bahrain. Variability within included studies was related to the covered lifestyle domain, mode of delivery of the intervention (either online, in person, or hybrid), and duration of the intervention (varying between hours and weeks). Ibrahim et al. concluded that the findings of the meta-analysis of included studies indicated the presence of improvement of knowledge, confidence, and practice, but insufficient power to measure the attitude. The current study did not measure confidence and practice of the participants but the improvement in the knowledge domain is similar to the findings of the study by Ibrahim et al. and lack of specific effect on attitude is similar to the findings of the current study, indicating that the developed interventions should invest more effort on changing attitudes rather than relying on personal factors related to knowledge, confidence, or skills.

In addition to implementing targeted educational interventions to enhance the knowledge, competencies, confidence, and skills of healthcare practitioners concerning LM counseling delivery, the recent global and local epidemiological situation and the prevalence of chronic NCDs indicated the importance of incorporating LM education in curricula of medical schools. This demand motivated the development and implementation of LM residency curricula in the US by the ACLM that can be incorporated into different specialties (14, 15). Nonetheless, it is possible to argue that implementing LM education may face challenges depending on the context. For example, Shiach et al. (16) conducted a curriculum review and mapping of UK medical schools, surveys of students, and an assessment of nutrition education in UK medical programs. The findings indicated a gap in the integration of nutrition education into the UK medical education system.

Similar to the international context, the Saudi Board for Family Medicine Curriculum 2024 has multiple competencies that target preventive and curative healthcare services, including the promotion of a healthy lifestyle, especially for patients at risk or suffering from chronic NCDs (17). Furthermore, the recent release of the SCFHS Lifestyle Medicine Fellowship Program is aligned with the principles of healthcare transformation and the development of the new Saudi Model of Care, which gives more emphasis on the utilization of evidence to improve healthcare service delivery (18) and the adoption of a healthy lifestyle on individual and community levels (19). This transformative context and the findings of the current study indicate the importance of promoting LM education within postgraduate family medicine training programs at the national level to further improve the attitude of the physician and their competency toward LM and to equip them to meet the demand associated with the ongoing transformation at the national level.

The findings of the current study can be compared to similar international investigations that have conducted medical education interventions in other clinical specialties. Among the recruited sample of residents, the difference in knowledge between pre- and post-increase was 82%, reflecting high effectiveness in medical education. This result concurs with a study about the effectiveness of interactive teaching intervention on medical students’ knowledge and attitudes toward stem cells, pre- and post-test design among 71 medical students in 6th year medical students at the University of Science and Technology Yemen Jordan branch, 2018–2019, which revealed total scores of stem cell-related knowledge and attitudes significantly improved post intervention (20). In agreement with a study using a pre-test/post-test quasi-experimental design among 200 students from a medical university in Iran regarding social determinants of health, post-test median scores were significantly elevated compared to pre-test knowledge scores (*z* = 11.89, *p* < 0.001) and attitude scores (*z* = −11.60, *p* < 0.001) (21). These two studies on different education topics indicated that educational intervention improved students’ knowledge; the same methodology indicated that medical education intervention contributes to increasing the level of knowledge among students and residents or different participants who were exposed to medical education. We advised this method to increase awareness and knowledge of the topics of interest among health professionals, thereby positively impacting patient care. Overall change in attitudes was considered low, at 56% improvement after intervention, compared to improvement in knowledge. The results disagree a little with a study conducted among medical students and trainees (third-year medical students before entering clerkship), in a teaching hospital in Tehran, where the education effectively shifted students’ attitudes toward system-based thinking and reinforced individual responsibility (22).

Although the current study was mainly developed to provide an assessment of the effectiveness of an educational intervention to enhance the knowledge and attitude of family medicine residents concerning LM in their family clinics, the findings present a plausible interpretation of how the limited improvement in the attitude of the physicians toward LM might influence optimum implementation of the newly developed Saudi Model of Care. This model shifts healthcare delivery from a curative, hospital-centric framework to a proactive system rooted in primary care and family medicine practices (23). Part of this shift is the delegation of lifestyle counseling to specialized roles, such as those in the “Keep Well” initiative (e.g., Health Coaches).

Nonetheless, it is possible to argue that the implementation of such initiatives might face resistance from family medicine physicians. This resistance can be linked to challenges in professional identity and role perception within medical education perspectives (24). Therefore, family physicians may view lifestyle interventions as secondary to clinical diagnosis. Subsequently, it is possible to argue that integrating LM education during early undergraduate medical school years is likely to enhance the professional attitude of the physician concerning LM. As a part of the Saudi Vision 2030, the Health Holding Company supports this by fostering an integrative ecosystem where these specialized roles ensure that lifestyle counseling is not an informal adjunct but a standardized pillar of the social, mental, and physical health framework (19, 25).

In addition to the importance of addressing resistance concerning the implementation of LM counseling in clinical settings during the early stage of medical education, it is important to understand factors associated with difficulties in changing family physicians’ behavior toward the counseling. In the current context, the COM-B model of behavior and the PRIME theory of motivation can be applied as a framework for understanding factors associated with the effectiveness of the intervention applied in the current study. The model addresses three factors associated with behavior: capability, opportunity, and motivation. Motivation is a core factor linked to the dynamic processes of planning and evaluation, and is associated with emotional and habitual perspectives that lead to eventual response (behavior) (26).

It is possible to argue that the current intervention addressed the psychological capacity among the residents by increasing their knowledge about LM counseling. However, the limited improvement in their attitude is linked to the limited influence on improving the reflective and automatic motivations associated with conscious evaluations, beliefs, and habitual responses. Finally, the residents may have perceived limited opportunities (either physical or social) to induce change due to their understanding of the limited cultural support in the clinical environment, lack of dedicated time or resources for LM counseling, or feeling that changing their practicing behavior is impossible. Eventually, the residents are likely to exhibit limited emotional and habitual perspectives with regard to the implementation of the LM counseling in their clinical settings, thus leading to marked resistance to change of attitude. This indicates that, to enhance the effectiveness of the intervention, more emphasis should be applied to motivating residents by addressing physical and social opportunities and not only resorting to psychological capability.

The current study targeted family medicine physicians to improve their LM counseling capabilities in a Saudi Arabian context. The implementation was motivated by the high demand imposed by the epidemiology of chronic NCDs, where family medicine physicians are likely to manage cases related to obesity, diabetes mellitus, hypertension, and dyslipidemia (27), which require competent behavioral modification skills. Since the rise in the incidence of chronic NCDs is a global issue, the educational LM intervention developed in the current study can be implemented in comparable training residency programs in other countries. For example, in a recent assessment of the burden of NCDs in South Asia, the incidence of diabetes increased by 21% between 2010 and 2021 (28). Similarly, a recent systematic review and meta-analysis that assessed the prevalence of cardiometabolic diseases, including diabetes, hypertension, stroke, hypercholesterolemia, and cardiovascular diseases, revealed a high prevalence of these conditions varying between 6 and 27% (29). These findings indicate the importance of transferring or adopting lifestyle educational interventions across other settings suffering from the rise of chronic NCDs, especially with limited financial resources directed toward curative healthcare services and to provide more concentration on preventive ones.

This study has multiple areas of strengths and limitations. The main strength of the study is its use an interventional approach to measure changes in the knowledge and attitudes of family medicine trainees regarding LM and how these changes affect their LM competency. However, there are multiple limitations related to the current investigation. First, the small sample size limits the generalizability of the study. However, it is possible to argue that the current findings indicate a reasonable positive impact on enhancing the competency of the family medicine residents with regard to LM and can form a basis for larger LM intervention programs. Additionally, the lack of a clear control group and the utilization of a pre- and post-approach is likely to be affected by test–retest familiarity, thus weakening the causality attribution to the intervention. Furthermore, the responses of the participants can be affected by social desirability, leading to the possibility of measurement bias. Finally, the intervention lacks a follow-up plan, which may lead to knowledge decay and undermine its effects. This indicates the importance of planning for follow-up at 3-, 6-, or 12-month intervals after the application of the intervention to ensure its sustainability.

## 5 Conclusion

Educational intervention led to a marked improvement in residents’ knowledge and a moderate enhancement in their attitudes toward LM. These findings emphasize the need for strengthening medical education in the principles of LM among family physicians, which is the specialty dealing with diseases related to behavior change in the successful management plan. We recommend the expanded outcome of this study to another study that measures current practices and training in the clinical application of LM prescription in family medicine clinics. The future application of the intervention at a larger national scale is recommended to be accompanied by an assessment of its effectiveness via a large-scale interventional study.

## Data Availability

The original contributions presented in the study are included in the article/supplementary material, further inquiries can be directed to the corresponding author.

## References

[1] Geng X Liang F Wang P. The global burden of non-communicable diseases attributable to behavioral risk factors and its trends from 1990 to 2021. J Adv Res. (2025). S2090-1232(25)00726-X. doi: 10.1016/j.jare.2025.09.022, 40957476 PMC13227288

[2] Aljarid S Alonazi W. Examining factors contributing to mortality in Saudi Arabia: proposing effective healthcare management approaches. BMC Public Health. (2025) 25:1801. doi: 10.1186/s12889-025-22421-z, 40375232 PMC12083158

[3] Alghnam S Bosaeed M Aljouie A Alshahrani SM Alshenqeety O Atun R . Estimating the prevalence of select non-communicable diseases in Saudi Arabia using a population-based sample: econometric analysis with natural language processing. Ann Saudi Med. (2024) 44:329–38. doi: 10.5144/0256-4947.2024.329 39368120 PMC11454953

[4] Arabia S. . (2019). World Health Survey Saudi Arabia (KSAWHS). Ministry of Health, Kingdom of Saudi Arabia Final report. Available online at: https://www.researchgate.net/publication/356665387_World_Health_Survey_Saudi_Arabia_2019

[5] Mohamed AH Darraj M Yassin A Somaili M Sayed A Oraibi O . Prevalence and short-term clinical impacts of new-onset diabetes mellitus among patients with COVID-19 in Jazan region, Saudi Arabia. BMC Endocr Disord. (2024) 24:1–12. doi: 10.1186/s12902-024-01724-z 39304825 PMC11414278

[6] Hakami SM Omar MT Alsaad SM Vennu VS Hattan LI Bindawas SM. Association between non-communicable diseases and physical activity level in older adults visiting primary health care centers in Jizan, Saudi Arabia. Saudi Medical Journal (2023) 44:580–7. doi: 10.15537/smj.2023.44.6.20230076 37343987 PMC10284233

[7] Grega ML Shalz JT Rosenfeld RM Bidwell JH Bonnet JP Bowman D . American College of Lifestyle Medicine Expert Consensus Statement: lifestyle medicine for optimal outcomes in primary care. Am J Lifestyle Med. (2023) 18:269–93. doi: 10.1177/15598276231202970, 38559790 PMC10979727

[8] Matthews JA Matthews S Faries MD Wolever RQ. Supporting sustainable health behavior change: the whole is greater than the sum of its parts. Mayo Clin Proc Innov Qual Outcomes. (2024) 8:263–75. doi: 10.1016/j.mayocpiqo.2023.10.002, 38807973 PMC11130595

[9] Ibrahim S Senff JR Sivakumar J Ventresca M Coulson J Singh S . Lifestyle Medicine Education in Health Professionals Curricula: A Systematic Review and Meta-Analysis. Am J Lifestyle Med. (2025) 31:15598276251362806. [Epub ahead of print]. doi: 10.1177/15598276251362806 PMC12313616 40756621

[10] Health Sector Transformation Program: Delivery Plan 2020–2021. (2021). Available online at: https://www.vision2030.gov.sa/media/u5xapka3/2021-2025-health-sector-transformation-program-delivery-plan-en.pdf (Accessed May 16, 2026).

[11] Saudi Commission for Health Specialties (SCFHS). Saudi Board for Family Medicine: Curriculum 2024. (2024). Available online at: https://scfhs.org.sa/sites/default/files/2024-12/FM%20CURRICULUM%202024-compressed.pdf (Accessed May 16, 2026).

[12] Saudi Commission for Health Specialties (SCFHS). Lifestyle Medicine Fellowship Program (2021). Available online at: https://scfhs.org.sa/sites/default/files/2022-02/Life%20style%20Med.pdf (Accessed May 16, 2026).

[13] Almansour M Salman A Id A Abdulaziz S Id A. State of lifestyle medicine education in Saudi medical schools: a descriptive study. Plos One. (2024) 19:e0308499. doi: 10.1371/journal.pone.0308499 39116151 PMC11309438

[14] Rea B Worthman S Shetty P Alexander M. Analytic Medical Education Transformation: Lifestyle Medicine in Undergraduate and Graduate Medical Education, Fellowship, and Continuing Medical Education. Thousand Oaks, CA: SAGE Publications. (2021). p. 1–12. 10.1177/15598276211006629 PMC8504331 34646100

[15] Miller CM Cramer SR Aney SJ Edmiston K Holmes S Mello S . (2025). Supplement: curriculum the innovation, implementation, and dissemination of the lifestyle medicine residency curriculum (LMRC) in graduate medical education. American Journal of Lifestyle Medicine, 19:10–20. doi: 10.1177/15598276251393211 PMC12646936 41311584

[16] Shiach A Thomson AC Samuels F. Persistent gaps in nutrition education in UK medical schools: a triangulated review of curricula, student perception and the evidence base. BMJ. (2026) :bmjnph-2025-001479. Available online at: https://nutrition.bmj.com/content/bmjnph/early/2026/04/20/bmjnph-2025-001479.full.pdf 10.1136/bmjnph-2025-001479 PMC13425111 42540135

[17] Saudi Commission for Health Specialties (SCFHS). Saudi Board for Family Medicine: Curriculum. Riyadh, Saudi Arabia (Alquorain): Saudi Commission for Health Specialties (SCFHS). (2024) 2:2024.

[18] Suleiman AK. Transforming healthcare: Saudi Arabia’s vision 2030 healthcare model. J Pharm Policy Pract. (2025) 18:1–20. doi: 10.1080/20523211.2024.2449051, 39845746 PMC11753010

[19] Chowdhury S Mok D Leenen L. Transformation of health care and the new model of care in Saudi Arabia: Kingdom’s Vision 2030. Journal of Medicine and Life. (2021) 14:347–54. doi: 10.25122/jml-2021-0070 34377200 PMC8321618

[20] Kap P. Development of valid and reliable questionnaire to evaluate knowledge, attitude, and practices (KAP) of lifestyle medicine domains. Healthcare J MDPI (2024) 12:1652. doi: 10.3390/healthcare12161652 PMC11353863 39201210

[21] Frates B Ortega HA Freeman KJ Co JPT Bernstein M. Lifestyle medicine in medical education: maximizing impact. Mayo Clin Proc Innov Qual Outcomes. (2024) 8:451–74. doi: 10.1016/j.mayocpiqo.2024.07.003, 39263429 PMC11387546

[22] Abdulrazeq F Kheirallah KA al-Mistarehi AH al Bashir S ALQudah MA Alzoubi A . Effectiveness of interactive teaching intervention on medical students’ knowledge and attitudes toward stem cells, their therapeutic uses, and potential research applications. PeerJ. (2022) 10:e12824. doi: 10.7717/peerj.12824, 35116201 PMC8785657

[23] Trad AA Al Daffalla NE Muabid EA Al Malibari GA. New model of care approach at primary health care. Journal of Clinical Nursing Reports (2023) 3, 1–5. Available online at: https://www.mkscienceset.com/articles_file/274-_article1734615480.pdf

[24] Monrouxe LV. Identity, identification and medical education: why should we care? Med Educ. (2010) 44:40–9. doi: 10.1111/j.1365-2923.2009.03440.x. 20078755, 20078755

[25] Health Holding Company. (n. d.) The Saudi model of care. Ministry of Health, Kingdom of Saudi Arabia. Available online at: https://www.health.sa/en/model-of-care (Accessed June, 10, 2026).

[26] West R Michie S (2020). A brief introduction to the COM-B model of behaviour and the PRIME theory of motivation. Qeios. 1–7.

[27] Pradhan J Pai M Dwivedi R Mishra B Behera S Bera T . Burden of non-communicable diseases in South Asia: a decomposition analysis. J Heal Popul Nutr. (2025) 44:12. doi: 10.1186/s41043-025-00827-0, 40251654 PMC12008954

[28] Cassambai S Tetteh J Highton P Kunutsor SK Darko O Jeffers S . Prevalence of cardiometabolic diseases in sub-Saharan Africa: a systematic review and meta-analysis review and meta-analysis. Glob Health Action. (2025) 18. doi: 10.1080/16549716.2025.2580758 PMC12616651 41229379

[29] Amini-Rarani M Omid A Nosratabadi M. The effect of an educational program on the knowledge and attitude of medical sciences students about social determinants of health in Iranian university students: a quasi-experimental study. Health Sci Rep. (2024). 7:e70182. doi: 10.1002/hsr2.70182, 39512255 PMC11540831

